# Revision of the Afrotropical genus *Leiodontocercus* (Orthoptera, Tettigoniidae, Phaneropterinae) with a description of four new species

**DOI:** 10.3897/zookeys.951.53814

**Published:** 2020-07-22

**Authors:** Bruno Massa

**Affiliations:** 1 Department of Agriculture, Food and Forest Sciences, University of Palermo, Italy University of Palermo Palermo Italy

**Keywords:** Central and West Africa, leaf katydids, new species, speciation, taxonomy

## Abstract

Specimens belonging to the genus *Leiodontocercus* are rare or even absent in natural history museum collections; this is likely due to at least two reasons, notably, their relatively small size, and, the sheer difficulty in finding them in dense Afrotropical forests. Until recently, three species from less than fifteen specimens were known from this genus, whose identification relied on a singular diagnostic character, that is, the shape of the male cerci. The present contribution is based on the examination of thirty specimens collected from various countries, ranging from central to west Africa; apart from the male cerci, a second diagnostic character – the stridulatory file – is used to distinguish species, even though it is difficult to examine in mounted specimens. As a result, four new species were detected, namely, *L.vicii***sp. nov.**, *L.spinicercatus***sp. nov.** (from the Central African Republic), *L.muticus***sp. nov.** (from Gabon and Cameroon) and *L.philipporum***sp. nov.** (from Côte d’Ivoire). Moreover, *L.condylus* is recorded from the Central African Republic, the only country where three species of this genus co-occur. It is suggested that population isolation during fluctuating humid and dry periods, consequent to the influence of Ice Age impact during the Pleistocene in tropical central Africa, is the best explanation for the adaptive radiation of the group.

## Introduction

The genus *Leiodontocercus* was described by Chopard (1954) together with its type-species *L.angustipennis* from Mt. Nimba (Guinea, tropical Africa). Ragge (1962), subsequently revised the genera of the tribe Phlaurocentrini Karsch, 1889, describing two new species within this genus, *L.condylus* from the Democratic Republic of Congo and *L.malleus* from Ghana; Ragge’s new descriptions were based on the shape of the male cerci. Since these initial works, very few specimens were studied: Chopard (1954) examined only one specimen, Ragge (1962) studied a further 12 specimens, while Massa (2013) examined another seven specimens, recorded from the Central African Republic; finally, Massa et al. (2020) listed 21 specimens. The present author studied a total of 30 specimens for this revision, most of which were collected during different entomological expeditions to the Côte d’Ivoire, Central African Republic, Cameroon and Gabon, respectively.

## Material and methods

The species currently grouped in *Leiodontocercus* were, until recently, recognized only by the shape of the male cerci; no other characters have hitherto been known or proposed to separate the species. In this paper, the stridulatory file under the male’s left forewing and the associated number and arrangement of teeth have been used as diagnostic characters. They are useful characters that determine whether species are bioacoustically separated from another one (Ragge 1980, Heller 2006).

Specimens studied for this contribution were collected at night time, attracted to a light trap (UV) that was set up both on the ground and in the canopy (35 to 55 meters high) in central-western countries of tropical Africa (Côte d’Ivoire, Gabon, Cameroon and the Central African Republic). Before mounting the specimens, the left wing of every male characterized by different cerci was spread in a manner that allowed a clear examination of the stridulatory file under the fore wing. Some specimens were dissected to inspect organs as well as to extract eggs from female specimens. Characters of specimens, stridulatory area, stridulatory file, cerci in frontal and lateral views were photographed with a Nikon Coolpix 4500 digital camera, mounted on a Wild M3 Stereomicroscope. Photographs were integrated using the freeware CombineZP (Hadley 2008). Mounted specimens were measured with a digital caliper (precision 0.01 mm); the following measurements were taken (in mm): body length: dorsal length from the head to the apex of the abdomen; pronotum length and height; tegmina: length and maximum width; hind femora length.

In view of the difficulty to distinguish between females of different species, in the present paper they are listed together with male specimens that were collected in the same locality and on the same date. For the same reason, no females are listed within the paratypes of new species, but merely as material examined. Thus, the description of female characters is reported within that of the genus.

**Abbreviations used in this paper**

**ANHRT** African Natural History Research Trust, Hereford, UK;

**BMPC** Bruno Massa Private Collection, Palermo, Italy;

**MNHN**Muséum National d’Histoire Naturelle, Paris, France;

**MSNP** Museo di Storia Naturale, University of Pavia, Italy;

**NHW** Naturhistorisches Museum Wien, Vienna, Austria;

**PAPC** Philippe Annoyer Private Collection, Sainte Croix Volvestre, France.

## Results

### Characters of *Leiodontocercus* Chopard, 1954 (species-type: *L.angustipennis* Chopard, 1954)

**Figures 1–3.**
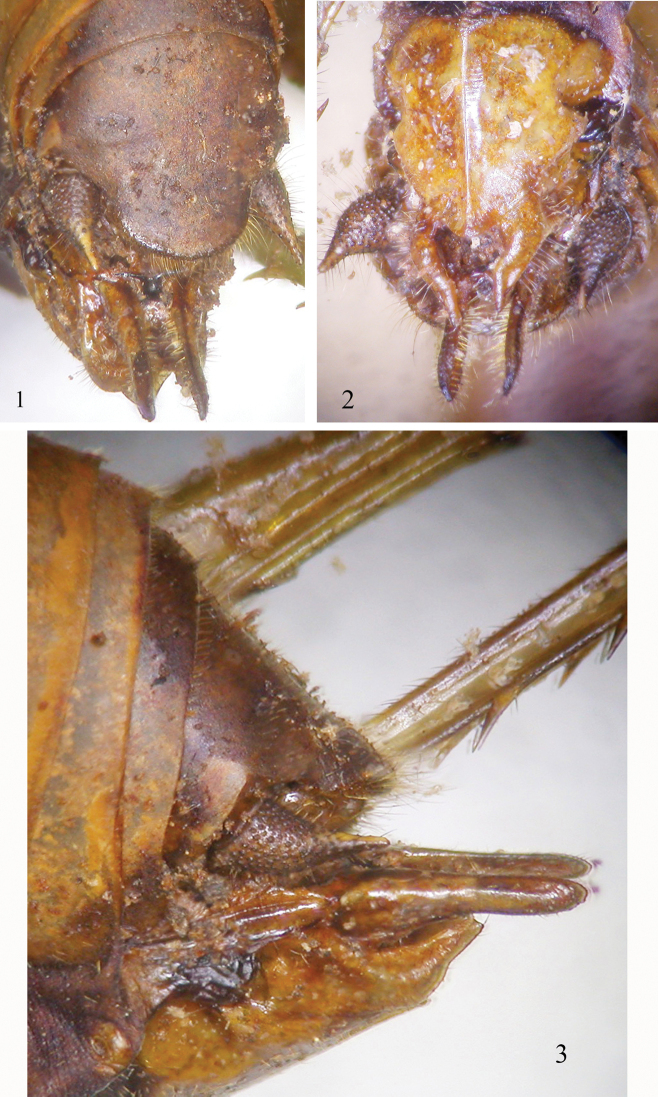
Female ovipositor of *Leiodontocercus*: **1** 10^th^ abdominal tergite concealing the supra-anal plate in the female of *L.muticus* sp. nov. from Gabon **2** ventral view of the ovipositor of *L.spinicercatus* sp. nov. from Central African Republic **3** lateral view of the ovipositor of *L.spinicercatus* sp. nov. from Central African Republic.

The word *Leiodontocercus* derives from the Greek and means “cercus with a smooth tooth” (λέιος = smooth, ὀδόντος genitive of ὀδούς = tooth). *Leiodontocercus* is characterized by a strongly compressed fastigium of vertex which slopes to the frons and is sulcate above; tegmina are very narrow, obliquely truncate apically; male last sternite without styli, and cerci stout and enlarged apically. Like in the other Phlaurocentrini, the 10^th^ abdominal tergite of the female is hood-like and conceals the supra-anal plate; the ovipositor is very similar to that of *Buettneria* Karsch, 1889, it is much reduced and with smooth and short valves. Ventral valves are short, upward and apically pointed, dorsal valves longer than ventral ones, straight like two short fingers; the subgenital plate of the female lacks diagnostic characters, in all specimens examined it is triangular with a central fine keel (Figs 1–3). Thus, like in the other genera of the tribe Phlaurocentrini the valves of the ovipositor are not flattened laterally. This indicates that the eggs are not inserted between the layers of the leaf epidermis, as in most Phaneropterinae, but possibly they are laid between cracks of tree bark. The eggs of *Leiodontocercus* species are not flat, like most species of Phaneropterinae, but nearly round and thick, similarly to species of *Phlaurocentrum* Karsch, 1889. Very likely this shape conveys a high resistance to desiccation (very thick chorionic layers that reduce the rate of water loss). The number of eggs found within the female oviduct was low (between 10 and 15).

### Annotated list of species

#### Leiodontocercus angustipennis

Taxon classificationAnimaliaOrthopteraTettigoniidae

Chopard, 1954

5DE7E087-3AC1-5BDC-8767-46886A4DF049

Figs 14 , 16 , 16a

Leiodontocercus angustipennis Chopard 1954. Mem. Inst. franc. Afr. Noire 40(2): 84; type locality: Mt. Nimba, Guinea (MNHN).

##### Material examined.

Guinea, Mt. Nimba (♂ holotypus) (MNHN)

##### Distribution.

After the description by Chopard (1954), Ragge (1962) recorded another specimen from Sierra Leone. Massa (2013) recorded *L.angustipennis* also from the Central African Republic, but later Massa et al. (2020) stated that the specimens were erroneously identified and actually they belong to *L.condylus*; in addition, they wrote that other specimens belong to another two undescribed species, described below.

#### Leiodontocercus philipporum sp. nov.

Taxon classificationAnimaliaOrthopteraTettigoniidae

60506368-AF8F-5A8F-AB0C-730D28841F3B

http://zoobank.org/18E7A412-0C64-44B7-808C-C250EA4479BF

Figs 1 , 7 , 12 , 19 , 20

##### Material examined.

Côte d’Ivoire, Lamto Nature Scientific Reserve, Bandama River, 4.IX.1982 (♂ holotypus) (BMPC); Côte d’Ivoire, Taï National Park, Research Station, 22.III–4.IV.2017, P. Moretto & P. Annoyer (3♀) (BMPC).

**Figures 4–8.**
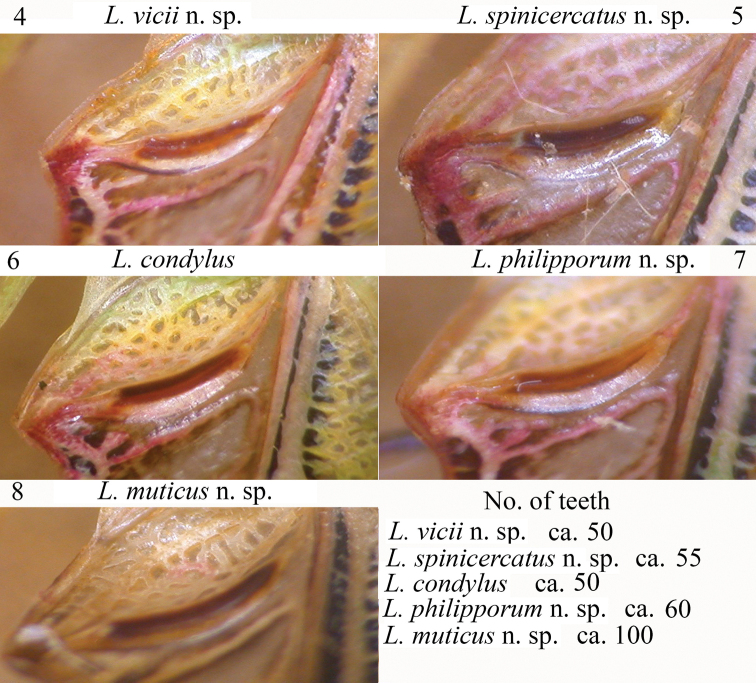
Stridulatory file and detail of teeth in the following species of *Leiodontocercus*: **4***L.vicii* sp. nov. **5***L.spinicercatus* sp. nov. **6***L.condylus***7***L.philipporum* sp. nov. **8***L.muticus* sp. nov.

##### Description.

**Male. *General habitus and colour.*** Predominantly green-brown, two lateral black spots on fore margin of pronotum and corresponding hind margin of head, black stripe interrupted on the hind margin of pronotum, abdomen yellow, last abdominal tergite orange, antennal segments reddish, legs yellowish, hind tibiae yellowish with black rings. ***Head and antennae.*** Eyes oval-roundish, prominent, antennae long and thin. ***Thorax.*** Anterior margin of pronotum slightly concave, posterior margin straight. Lower margin of pronotal lobes rounded. Tegmina very narrow. Central part of the stridulatory file consists of ca 60 teeth (Fig. 7). The stridulatory area of the left tegmen wider than the rest of tegmen (Fig. 12). Right tegmen without mirror. ***Legs.*** Fore coxae armed. Tympana on fore tibiae open on outer, closed on inner side. Fore femora with 8 inner ventral spines, fore tibiae with 4 inner and outer ventral spines. Mid femora armed with 7 outer ventral spines, mid tibiae dorsally with 2 inner spines, 7 spines on outer and inner ventral margins. Hind femora with 8–9 outer and inner ventral spines, hind tibiae straight with many ventral spines. Two pairs of small spines on the outer and inner knees of hind femora. ***Abdomen.*** Cerci stout and hairy, in frontal view apically triangular with serrated margins; ventrally they have a long-tipped appendage (Figs 19, 20). Hind margin of the subgenital plate nearly straight, styli absent.

##### Measurements (mm).

Body length: 19.4; length of pronotum: 3.0; depth of pronotum: 3.2; length of hind femora: 20.0; length of tegmina: 24.4; width of tegmina: 3.3.

##### Etymology.

*Leiodontocercusphilipporum* sp. nov. is named after Philippe Annoyer and Philippe Moretto, who organized a one-month entomological mission to Taï National Park and Mt. Tonkoui of the Côte d’Ivoire, helping me in the night trapping and generously providing all Orthoptera collected there.

#### Leiodontocercus spinicercatus sp. nov.

Taxon classificationAnimaliaOrthopteraTettigoniidae

E158164E-1C9A-5DD4-B10D-B94250568FDF

http://zoobank.org/F34AB84D-BFF9-4505-8CCE-C35AAB07FCCE

Figs 2 , 3 , 5 , 10 , 23 , 24

##### Material examined.

Central African Republic, Dzanga-Sangha Special Reserve, Camp 5, 15–16.II.2005, P. Annoyer (♂ holotypus); Dzanga-Sangha Special Reserve, Camp 5, 7–8.II.2005 (light), P. Annoyer (1♀); Dzanga-Sangha Special Reserve, 15–16.X.2008 (light), P. Annoyer (1♀) (BMPC).

##### Description.

**Male. *General habitus and colour.*** Predominantly green-brown, two lateral black spots on fore margin of pronotum and corresponding hind margin of head, black stripe interrupted on the hind margin of pronotum, abdomen yellow, last abdominal tergite orange, antennal segments reddish, legs yellowish, hind tibiae yellowish with black rings. ***Head and antennae.*** Eyes oval-roundish, prominent, antennae long and thin. ***Thorax.*** Anterior margin of pronotum slightly concave, posterior margin straight. Lower margin of pronotal lobes rounded. ***Tegmina*** very narrow. Central part of the stridulatory file consists of ca 55 teeth (Fig. 5). The stridulatory area of the left tegmen wider than the rest of tegmen (Fig. 10). Right tegmen without mirror. ***Legs.*** Fore coxae armed. Tympana on fore tibiae open on outer, closed on inner side. Fore femora with 7 inner ventral spines, fore tibiae with 4 inner and outer ventral spines. Mid femora armed with 9 outer ventral spines, mid tibiae dorsally with 2 inner spines, 7 spines on outer and inner ventral margins. Hind femora with 8–9 outer and inner ventral spines, hind tibiae straight with many ventral spines. 2 pairs of small spines on the outer and inner knees of hind femora. ***Abdomen.*** Cerci stout and apically incurved, with an apical ventral pointed tip (Figs 23, 24). Hind margin of the subgenital plate nearly straight, styli absent.

**Figures 9–14.**
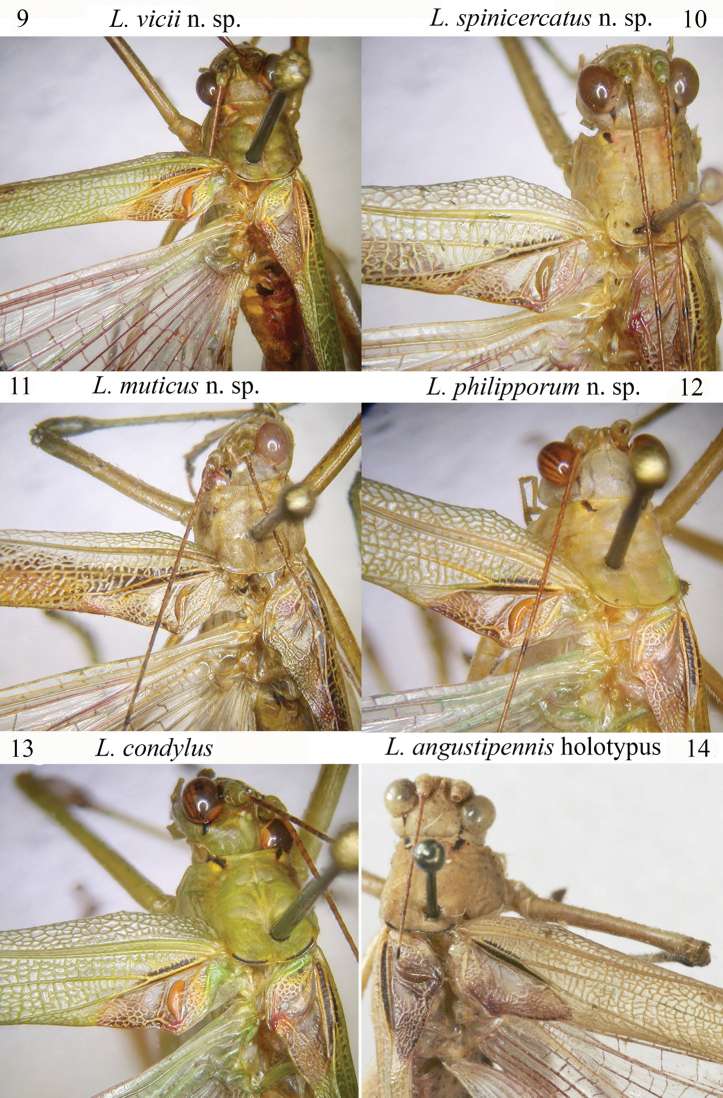
Dorsal view of the head, pronotum and the stridulatory area of the following species of *Leiodontocercus*: **9***L.vicii* sp. nov. **10***L.spinicercatus* sp. nov. **11***L.muticus* sp. nov. **12***L.philipporum* sp. nov. **13***L.condylus***14***L.angustipennis* (holotypus).

##### Measurements (mm).

Body length: 14.2; length of pronotum: 2.9; depth of pronotum: 2.5; length of hind femora: 19.7; length of tegmina: 23.4; width of tegmina: 3.2.

##### Etymology.

*Leiodontocercusspinicercatus* sp. nov. is named after the ventral spine on the male cerci.

##### Distribution.

It is known from the Dzanga-Sangha Special Reserve (Central African Republic).

#### Leiodontocercus vicii sp. nov.

Taxon classificationAnimaliaOrthopteraTettigoniidae

2C358D8E-DBF0-5586-85FC-925A91656E64

http://zoobank.org/6F371136-432D-4F01-B6AD-4FDDC631CB2C

Figs 4 , 9 , 21 , 22

##### Material examined.

Central African Republic, Dzanga-Ndoki NP, Lake 1, 8–10.II.2012, SANGHA2012 Team (♂ holotypus) (BMPC); Dzanga-Ndoki NP, Lake 1, 20–23.II.2012 (hand catching and light), SANGHA2012 Team (1♂ paratypus) (BMPC).

##### Description.

**Male. *General habitus and colour.*** Predominantly green-brown, two lateral black spots on anterior margin of pronotum and corresponding hind margin of head, black stripe interrupted on the posterior margin of pronotum, abdomen yellow, last abdominal tergite orange, antennal segments reddish, legs yellowish, hind tibiae yellowish with black rings. ***Head and antennae.*** Eyes oval-roundish, prominent, antennae long and thin. ***Thorax.*** Anterior margin of pronotum slightly concave, posterior margin straight. Lower margin of pronotal lobes rounded. ***Tegmina*** very narrow. Central part of the stridulatory file consists of ca 50 teeth (Fig. 4). The stridulatory area of the left tegmen less protruding backwards than in the other species (Fig. 9). Right tegmen without mirror. ***Legs.*** Fore coxae armed. Tympana on fore tibiae open on outer, closed on inner side. Fore femora with 8 inner ventral spines, fore tibiae with 4 inner and outer ventral spines. Mid femora armed with 7 outer ventral spines, mid tibiae dorsally with 2 inner spines, 7 spines on outer and inner ventral margins. Hind femora with 7–8 outer and inner ventral spines, hind tibiae straight with many ventral spines. 2 pairs of small spines on the outer and inner knees of hind femora. ***Abdomen.*** Cerci stout and apically swollen, with the apex down curved and its margins serrated (Figs 21, 22). Posterior margin of the subgenital plate nearly straight, styli absent.

**Female.** Unknown.

##### Measurements (mm).

Body length: 13.5–15.5; length of pronotum: 3.1–3.2; depth of pronotum: 2.8–2.9; length of hind femora: 20.0–20.1; length of tegmina: 24.7–24.8; width of tegmina: 2.3–2.4.

##### Etymology.

*Leiodontocercusvicii* sp. nov. is named after the nickname of my son-in-law Vincenzo Cigna, as sign of his esteem and sincere friendship.

##### Distribution.

Presently it is only known from the Dzanga-Ndoki National Park (Central African Republic).

#### Leiodontocercus condylus

Taxon classificationAnimaliaOrthopteraTettigoniidae

Ragge, 1962

F2FB972F-4C1F-5EDE-AB38-4565082164C7

**Figures 15–20.**
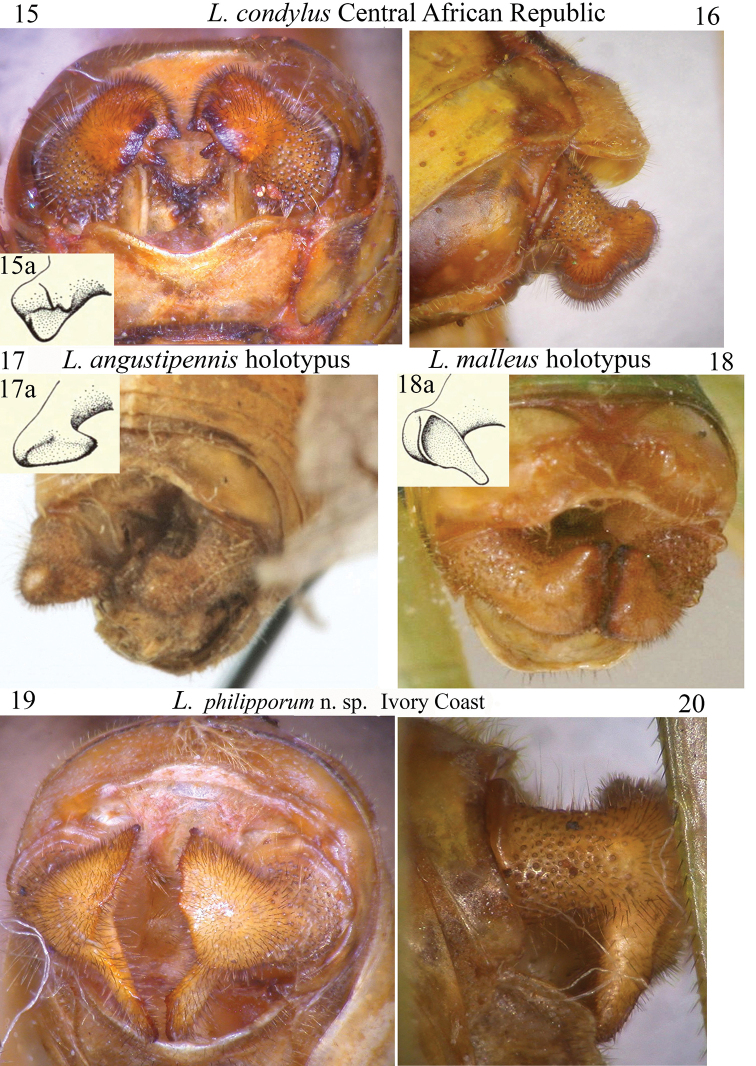
Frontal and lateral view of cerci of the following species of *Leiodontocercus*: **15, 16***L.condylus* (inset 15a: cercus after Ragge 1962), **17***L.angustipennis* (holotypus; inset **17a** cercus after Ragge 1962) **18***L.malleus* (holotypus; inset **18a** cercus after Ragge 1962) **19, 20***L.philipporum* sp. nov. **17** and **18** after OSFonline (http://orthoptera.speciesfile.org/Common/basic/Taxa.aspx?TaxonNameID=1136208).

Figs 6 , 13 , 15 , 15a , 16 , 27

Leiodontocercus condylus Ragge. 1962. Bull. Br. Mus. (Nat. Hist.) Ent. 13: 15; type locality: Kibali-Ituri, Yindi (Democratic Republic of Congo) (NHM).

##### Material examined.

Central African Republic, Dzanga-Ndoki National Park, Dieké 25.XI.2010, P. Annoyer (1♂, 1♀); Dzanga-Ndoki National Park, Lake 1, 31.I–2.II.2012 (1♀), 12–13.II.2012 (1♂), 13–14.II.2012 (3♂), 17.II.2012 (1♀); 20–23.II.2012 (1♀), 22–23.II.2012 (1♀); 28–29.II.2012 (1♂) (hand catching and light), SANGHA2012 Team; Lake 3, 25–26.II.2012 (light), P. Annoyer (1♀) (BMPC & PAPC); Central African Republic, La Maboké, M’Baiki II.1964, M. Pavan (1♂) (MSNP).

##### Remarks.

*Leiodontocercuscondylus* has the central part of the stridulatory file with ca 50 thick teeth, that appear just deeper than in the other species (Fig. 6). The stridulatory area of the left tegmen is a little backwards protruding, more than in the other species (Fig. 13). This species is characterized by cerci stout with an apical swelling with the outer margin serrated and the inner part with two pointed black tipped teeth (Figs 15, 16).

##### Distribution.

*Leiodontocercuscondylus* has been described from Zaire (= Democratic Republic of Congo) and has been reported from Central African Republic (Dzanga-Ndoki National Park) by Massa et al. (2020); it is here recorded also from the M’Baiki forest in Central African Republic. Presently females are not recognized at species level and were identified as *L.condylus* because they were collected together with the males of this species.

#### Leiodontocercus muticus sp. nov.

Taxon classificationAnimaliaOrthopteraTettigoniidae

64EABD67-B66A-54C8-86DC-033C676B9F8C

http://zoobank.org/10E50D8E-F830-4D8D-B231-A74919255CB5

Figs 8 , 11 , 25 , 26

##### Material examined.

Gabon, Mikongo (Rougier), Mts de Cristal (secondary forest) (430 m) 0°29'47"N, 11°10'42"E, 28.VII–12.VIII.2019 (MV Light Trap), Albert, Aristophanous, Bie Mba, Dérozier, Moretto (♂ holotypus, 1♂ paratypus) (ANHRT); Gabon, Mikongo (Rougier), Mts de Cristal (secondary forest) (430 m) 0°29'47"N, 11°10'42"E, 28.VII–12.VIII.2019 (Actinic Light Trap), Albert, Aristophanous, Bie Mba, Dérozier, Moretto (1♀) (ANHRT); Gabon, Nyonié (lowland forest) 0°2'22"S, 9°20'25"E (10 m) 23–28.VIII.2019 (LepiLED Light Trap), Albert, Aristophanous, Bie Mba, Dérozier, Moretto (1♂ paratypus) (BMPC); Gabon, Lope National Park 4.IV.2014 (light), N. Moulin (1♀) (BMPC); Cameroon, Campo Ma’an National Park (lowland rainforest) (950 m) 10–22.III.2018 (MV Light Trap), Fotsing, Ishmael, Miles, Safian (1♂ paratypus, 1♀) (ANHRT); Cameroon, Mundame (1♀) (NHW).

##### Description.

**Male. *General habitus and colour.*** Green-brown, tegmina brownish, abdomen yellow, last abdominal tergite brown, cerci brown, antennal segments reddish, legs yellowish. ***Head and antennae.*** Eyes oval-roundish, prominent, antennae long and thin. ***Thorax.*** Anterior margin of pronotum slightly concave, posterior margin rounded. Lower margin of pronotal lobes rounded. ***Tegmina*** very narrow. Central part of the stridulatory file consists of ca 100 teeth (Fig. 8). The stridulatory area of the left tegmen wider than the rest of tegmen (Fig. 11). Mirror absent on the right tegmen. ***Legs.*** Fore coxae armed. Tympana on fore tibiae open on outer, closed on inner side. Fore femora with 9 inner ventral spines, fore tibiae with 6 inner and outer ventral spines. Mid femora armed with 8 outer ventral spines, mid tibiae dorsally with 2 inner spines, 6 spines on outer and inner ventral margins. Hind femora with 9–10 outer and inner ventral spines, hind tibiae straight with many ventral spines. 2 pairs of small spines on the outer and inner knees of hind femora. ***Abdomen.*** Cerci stout and hairy, in frontal view slightly incurved with an apical bulge just serrated on inner margin (Figs 25, 26). Posterior margin of the subgenital plate nearly straight, styli absent.

**Figures 21–26.**
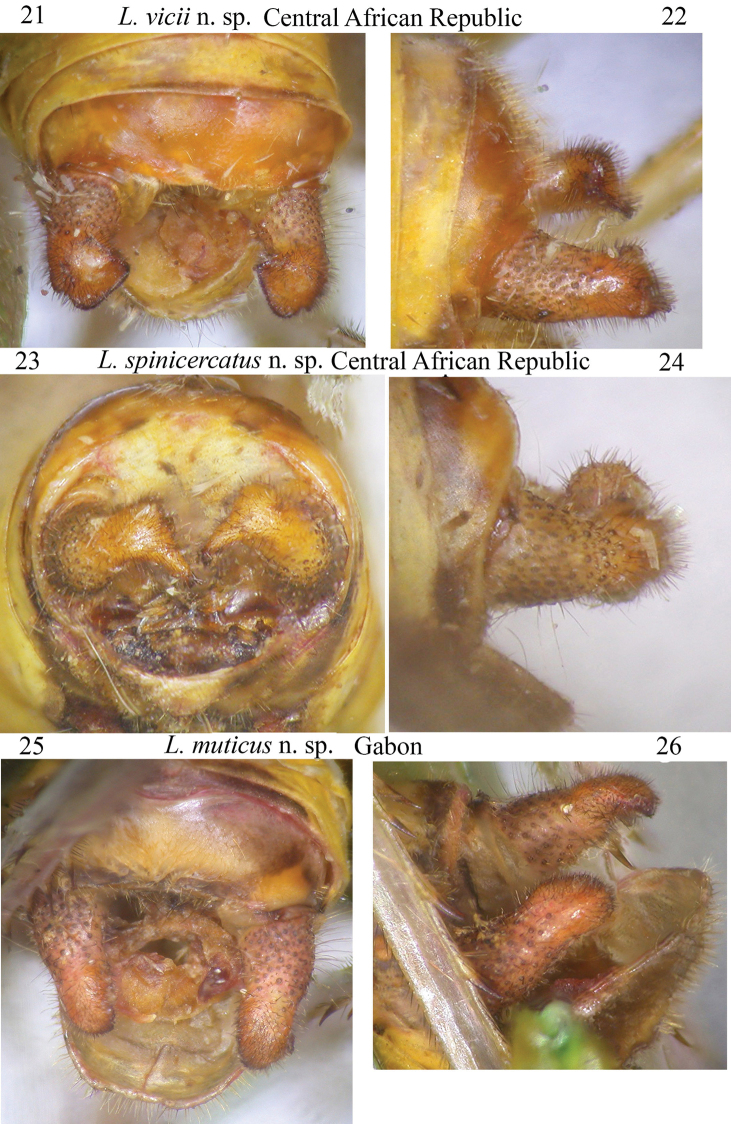
Frontal and lateral view of cerci of the following species of *Leiodontocercus*: **21, 22***L.vicii* sp. nov. **23, 24***L.spinicercatus* sp. nov. **25, 26***L.muticus* sp. nov.

**Figure 27.**
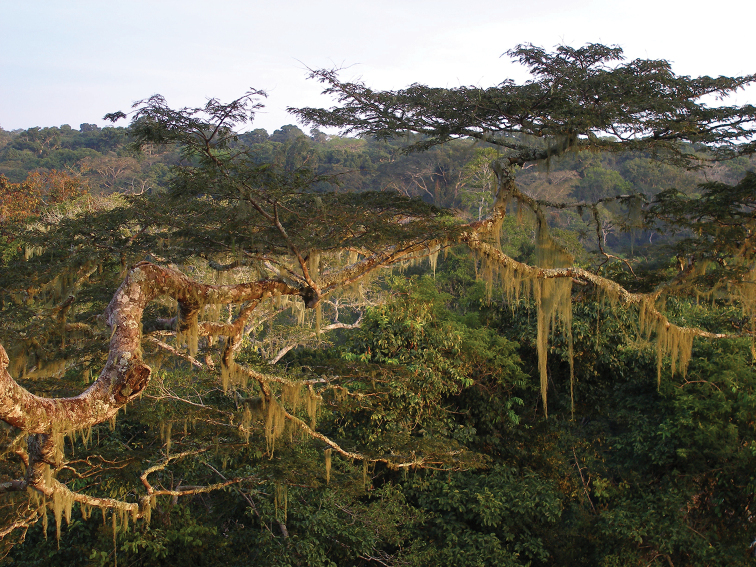
Multi-stratified canopy of the forest of the Dzanga-Ndoki National Park (Central African Republic), where *Leiodontocercus* species may occur exploiting different ecological niches (Photo by P. Annoyer).

**Female.** Interestingly, the females collected with males of *L.muticus* sp. nov. have black spots on the pronotum and black rings on the hind legs, like the other species of the genus. In addition, an alive female specimen photographed by P. Moretto (Fig. 29) shows alternate black and white abdominal sternites. In the males of *L.muticus* sp. nov. these black markings are absent. The female from Mundame (Cameroon) at NHW is tentatively identified, in absence of males.

**Figures 28, 29.**
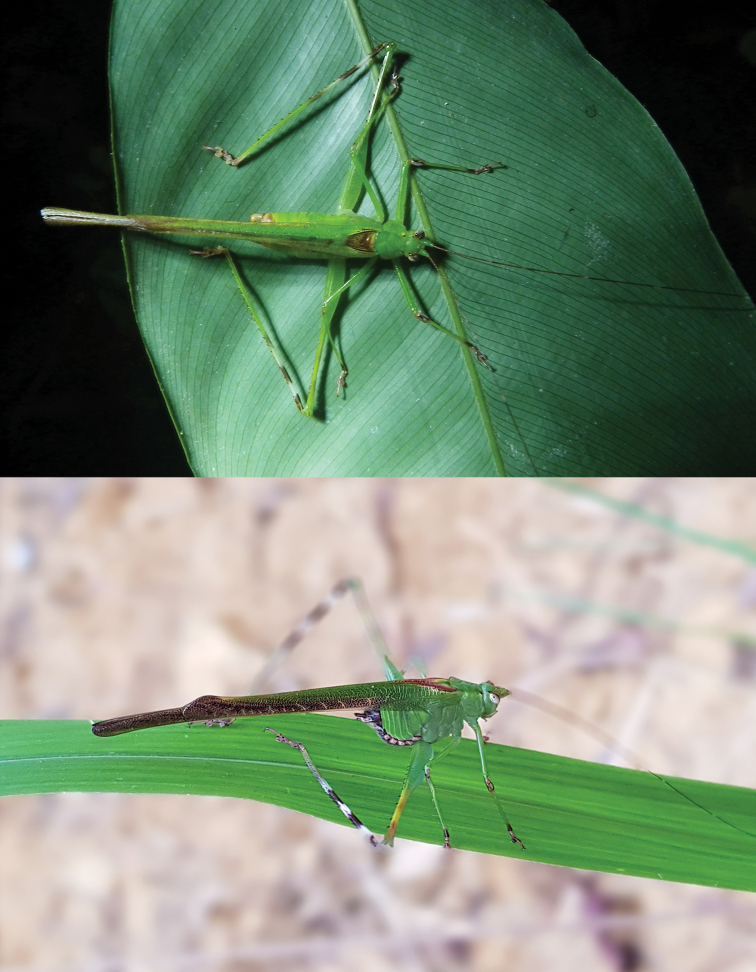
Mimicry of *Leiodontocercuscondylus* Ragge, 1962, Central African Republic, Dzanga-Ndoki National Park, 25^th^ November 2010 (Photo by S. Danflous) (above) and of *L.muticus* sp. nov., Gabon, Mikongo, 12^th^ August 2019 (Photo by P. Moretto) (below).

##### Measurements (mm).

Body length: 12.9–14.9; length of pronotum: 2.9–3.2; depth of pronotum: 3.0–3.2; length of hind femora: 19.6–19.7; length of tegmina: 24.4–24.6; width of tegmina: 3.4–3.5.

##### Etymology.

*Leiodontocercusmuticus* sp. nov. is named after the complete absence of any spine or appendage on the male cerci.

##### Distribution.

This species is known from some forested areas in Gabon and in Cameroon, situated about 300 km apart.

#### Leiodontocercus malleus

Taxon classificationAnimaliaOrthopteraTettigoniidae

Ragge, 1962

CD90599F-2F26-5FE0-8185-F2D250A50CB4

Fig. 18 , 18a

Leiodontocercus malleus Ragge. 1962. Bull. Br. Mus. (Nat. Hist.) Ent. 13: 14; type locality: Western Region, near Wiawso (Ghana) (NHM).

##### Notes.

This species is presently known only from the male holotype, another male paratype from Tafo (Ghana) and one female paratype from Ashanti (Ghana), localities not far to the north-east of Wiawso. Cerci are shown in Fig. 18 and 18a.

## Discussion

### The structure of the stridulatory file

The song produced by species of this genus is, to date, still unknown. Nonetheless, the stridulatory file (the structure that allows most Orthoptera to produce a song) was examined in detail for any discernable morphological differences. All the species of *Leiodontocercus* have a very short stridulatory file under the male’s left forewing, no longer than 0.5 mm (Figs 4–8). At first glance, even at very high magnification, it appears identical in all the males. However, a closer and more detailed examination revealed differences in the number of teeth, but not in their arrangement. The stridulatory file consists of very thick central teeth, that vary in number in the different species; in addition, the distal and the proximal parts of the stridulatory file have a small number of evenly-spaced small teeth. Evidently, the difference in the number of teeth (even though their structural arrangement is similar) and their different depth will produce a different song, which permits the male to attract a female of the same species. Among the examined species, the highest number of teeth in the central part of the stridulatory file (ca 100) has been found in *L.muticus* sp. nov., while a lowest number (ca 50) has been found in *L.condylus*, *L.spinicercatus* sp. nov. and *L.vicii* sp. nov., and an intermediate number (ca 60) was noted in *L.philipporum* sp. nov. Very likely, the song is produced using both central, distal and proximal teeth. It is remarkable to note that the three species with similar stridulatory files co-occur in the same areas of Central African Republic.

### The stridulatory area

The left and right tegmina of males bear the stridulatory area; this body portion is generally well characterized for each species. However, species of *Leiodontocercus* do not show great differences: the right forewing lacks the characteristic mirror, while the left forewing has an evident arched bulge that corresponds to the stridulatory file under the wing (Figs 9–14). Small diagnostic characters are recognizable in the different taxa: *L.vicii* sp. nov. has the left tegmen particularly narrow also in the stridulatory area, while the other species described in the present work have a deeper stridulatory area compared with the rest of the wing. In addition, *L.muticus* sp. nov., the species with the highest number of teeth in the stridulatory file, has a matching area on the dorsal left tegmen that is longer compared to other species. Furthermore, *L.condylus* has a stridulatory area that protrudes further backwards than that of the other species, while *L.malleus* has a brownish stridulatory area, sharply contrasting with the rest of the green-coloured tegmina (Ragge 1962); this brownish stridulatory area may also be observed in *L.condylus*.

### The shape of male cerci

The shape of the male cerci is the best diagnostic character of this genus; currently three different species have been described on the basis of the different cerci, and further, four new species are here described, mainly based on the shape of the cerci. The best way to observe cerci is through frontal and lateral views (Figs 15–26); this allows visibility of a possible ventral appendage, not otherwise visible through a dorsal view. Cerci are used by males during mating; many species of Tettigoniidae have been observed to use their cerci as a pincer that immobilizes the female’s ovipositor or abdomen (e.g., Vahed et al. 2014). It is highly likely that the shape of the male cerci and mating modality are congruent and that a female would recognize the male of the same species by the song it emits. Thus, we may presume that the female reacts to the stimulus originating from the song of a male of the same species and, subsequently, the male cerci could act as a second stimulus during mating.

### Habitat and habits of *Leiodontocercus* species

The Guineo-Congolian region, the tropical forest region of Central and West Africa, covers about 90% of the total forest surface in central Africa, but merely 6% in West Africa (Malhi et al. 2013). Most species of tropical African Tettigoniidae live in the multi-stratified canopy, and are nocturnal. All the species of *Leiodontocercus* have been found (generally single individuals) in multi-stratified and well-preserved primary forests (Fig. 27), and, in some cases, in secondary forests.

One live male specimen of *L.condylus* was photographed by Samuel Danflous in the Central African Republic (Dzanga-Ndoki National Park) in 2010 and one female of *L.muticus* sp. nov. was photographed by Philippe Moretto in Gabon (Figs 28, 29). They show a peculiar leg posture, with the femora more or less vertically positioned in respect to the body, similar to the posture of a spider or some grasshoppers of the Eumastacidae (C. Hemp, pers. comm.). The dark rings on hind tibiae, when exposed (as in these cases), and dark spots and markings on the body (including the abdomen of the female), may function in disruptive mimicry. Dark markings on the wings or on the legs are common within those species occurring inside the canopy (e.g., *Enochleticaostentatrix* Karsch, 1896, *Myllocentrum* species, some *Arantia* and *Eurycorypha* species, among others); it is very likely an adaptation to minimise predation by birds or other forest vertebrates, as well as invertebrates through mimicry.

### Speciation in *Leiodontocercus*

**Figure 30.**
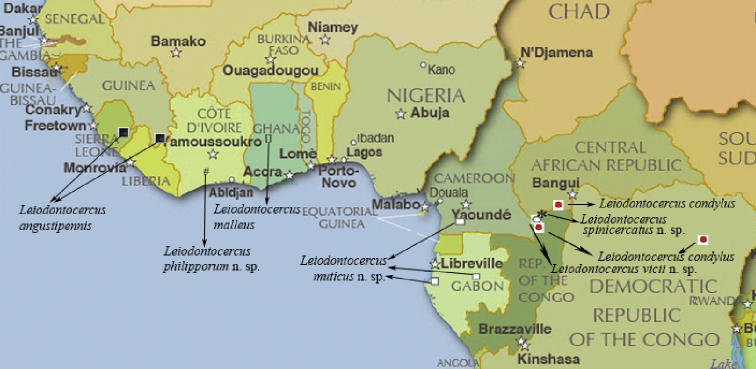
Updated distribution of the seven currently known species of the genus *Leiodontocercus*.

*Leiodontocercus* specimens are scarce in museums and collections, and this is probably the reason why their diversity has not been appreciated earlier. In addition, the species belonging to this genus are very small and delicate, with a body length that does not exceed 20 mm (15 mm on average) and a stridulatory file of no more than 0.5 mm; this makes it all the more difficult to study the very few existing, previously mounted specimens, accurately. Figure 30 shows the distribution of the currently known seven species of *Leiodontocercus*; interestingly, only the Central African Republic (protected areas Dzanga-Ndoki and Dzanga-Sangha) holds three species, which very likely occur syntopically. This finding probably results from more intensive research carried out in those areas, mainly through the use of light traps (UV), both on the forest floor and in the canopy (35 to 55 meters high) (cf. Massa 2013, Massa et al. 2020). The co-occurrence of different species distinguished by their different songs, different courtship behaviour, and small morphological differences including male cerci indicates the existence of reproductive barriers between them.

The high local biodiversity in central-western tropical African forests is shown by the high number of species of insects, Orthoptera being a case point. Generally, African Phaneropterinae are considered a taxonomic group with a great propensity to speciate; probably it is the forest ecosystem that facilitated speciation of most African Phaneropterinae. *Leiodontocercus* species, under a selective regime, may have acquired advantageous traits, that have increased local differentiation rate (cf. Simões et al. 2016). The case of speciation in *Leiodontocercus* is similar to that of *Tetraconcha* Karsch, 1890 (Phaneropterinae, Otiaphysini) (Massa 2017). Both genera show multiple speciation within tropical forest ecosystems of central and western Africa. Concerning *Leiodontocercus*, the small morphological disparity is very likely the effect of an evolutionary radiation, which may depend on local isolation. In the case of *Tetraconcha*, the morphological character observed to distinguish species is the stridulatory system, and in the case of *Leiodontocercus*, the main differences lie in the shape of the male cerci. Both the stridulatory system and cerci shape are linked to courtship and mating.

Climatic radiation is a type of geographic radiation in which allopatric speciation in the region is driven by changes in climate (Simões et al. 2016). In accordance with Maley (1996), African rainforests retreated during dry periods after the Ice Age, and climate fluctuations would have favored the dispersion of species. The climate of tropical Africa following the Ice Age was warmer and wetter than present (African humid period; Willis et al. 2013); in most Central African areas it shifted to a drier regime between 4000 and 2000 years BP, when the forest cover retreated (Willis et al. 2013). This may have allowed local isolation of populations that evolved in the absence of gene flow. Speciation events are often correlated with humid and dry periods; forest expansion during humid periods and retraction during dry periods are considered the best explanation for the patterns of geographical species distribution found on East African mountains (Schultz et al. 2007, Hemp et al. 2015); this climatic episode has also been proposed for *Tetraconcha* by Massa (2017) and is here proposed also for *Leiodontocercus*.

## Supplementary Material

XML Treatment for Leiodontocercus angustipennis

XML Treatment for Leiodontocercus philipporum

XML Treatment for Leiodontocercus spinicercatus

XML Treatment for Leiodontocercus vicii

XML Treatment for Leiodontocercus condylus

XML Treatment for Leiodontocercus muticus

XML Treatment for Leiodontocercus malleus
